# Selenium Status in Diet Affects Nephrotoxicity Induced by Cisplatin in Mice

**DOI:** 10.3390/antiox11061141

**Published:** 2022-06-10

**Authors:** Shuang Liu, Xing Wen, Qihan Huang, Minghui Zhu, Jun Lu

**Affiliations:** Key Laboratory of Luminescence Analysis and Molecular Sensing, Ministry of Education, College of Pharmaceutical Sciences, Southwest University, Chongqing 400715, China; liushuang_2022@163.com (S.L.); wenxing20222022@163.com (X.W.); huangqihan17@163.com (Q.H.); minghuizhu@163.com (M.Z.)

**Keywords:** cisplatin, nephrotoxicity, oxidative stress, thioredoxin, selenium, selenoprotein

## Abstract

Cisplatin is one of the most active chemotherapy drugs to treat solid tumors. However, it also causes various side effects, especially nephrotoxicity, in which oxidative stress plays critical roles. Our previous studies found that cisplatin selectively inhibited selenoenzyme thioredoxin reductase1 (TrxR1) in the kidney at an early stage and, subsequently, induced the activation of Nrf2. However, the effects of selenium on cisplatin-induced nephrotoxicity are still unclear. In this study, we established mice models with different selenium intake levels to explore the effects of selenoenzyme activity changes on cisplatin-induced nephrotoxicity. Results showed that feeding with a selenium-deficient diet sensitize the mice to cisplatin-induced damage, whereas selenium supplementation increased the activities of selenoenzymes TrxR and glutathione peroxidase (GPx), changed the renal cellular redox environment to a reduced state, and exhibited protective effects. These results demonstrated the correlation of selenoenzymes with cisplatin-induced side effects and provided a basis for the potential approach to alleviate cisplatin-induced renal injury.

## 1. Introduction

Cisplatin, chemically named cis-diamminedichloroplatinum (DDP), is a well-known chemotherapeutic drug [1]. It is a commonly used anticancer drug in clinic, which has been widely utilized in the treatment of cervical, head and neck, bladder, breast, blood, lung, testicular, ovarian, and esophageal cancers [2,3,4,5,6,7,8,9]. Platinum compounds, including cisplatin, are considered as cytotoxic drugs that kill cancer cells by damaging DNA, and inhibiting DNA synthesis and mitosis to induce apoptosis [1,10,11]. Although cisplatin has become the most widely used drug in clinical treatments of various solid tumors, its use has been largely limited due to the severe toxic side effects. There are approximately 40 specific toxicities of cisplatin, including nephrotoxicity, ototoxicity, hepatotoxicity, cardiotoxicity, gastrointestinal toxicity, neurotoxicity, and hematological toxicity. Among these side effects, nephrotoxicity is the most common one [6,12,13,14,15]. It has been reported that oxidative damage caused by excessive reactive oxygen species (ROS) is one of the main causes of cisplatin nephrotoxicity [16,17,18]. Long-term stress and hypoxia damage of the endoplasmic reticulum (ER) induced by cisplatin can lead to caspase-mediated apoptosis and the production of ROS, thus resulting in oxidative stress [19]. What is more, cisplatin may induce ROS production in microsomes by activating the cytochrome P450 system, thereby causing oxidative stress in proximal renal tubular epithelial cells (PTECs) [20,21].

Oxidative stress is an important marker of cisplatin-induced toxicity and is characterized by an imbalance between ROS production and clearance, which is regulated by antioxidant defense systems [22]. The thioredoxin (Trx) system and glutathione (GSH) system are two main important thiol-dependent antioxidant systems. The Trx system consists of nicotinamide adenine dinucleotide phosphate (NADPH), thioredoxin reductase (TrxR), thioredoxin (Trx), and peroxiredoxin (Prx) including cytoplasmic TrxR1, Trx1, and Prx 1 and 2 and mitochondrial TrxR2, Trx2 and Prx3. The GSH system is composed of NADPH, glutathione reductase (GR), GSH, and glutaredoxin (Grx)/glutathione peroxidase (GPx). These two systems make use of NADPH as the electron donor and transfer electrons to the substrate through various components in the system to remove ROS [23,24]. In addition, the Trx system also regulates the activity of many proteins related to cell death and oxidative stress, such as nuclear factor E2-related factor 2 (Nrf2), tumor suppressor protein 53 (p53), apoptosis-inducing factor (AIF), apoptosis signal-regulating kinase 1 (ASK1), etc. [25]. In the two systems, TrxRs and GPxs are the two major types of selenoenzymes in mammalian cells containing selenium in their active sites.

Selenium is an essential micronutrient for humans and animals. It participates in selenoprotein synthesis mainly through conversion to selenocysteine (Sec, U). Sec is known as the 21st amino acid, which is encoded by the codon TGA in DNA, and the corresponding stop codon UGA in mRNA [26,27,28]. In human cells, 25 selenoprotein genes have been identified [28]. Most selenoproteins including TrxR, GPx, and iodothyronine deiodinases (DIO) are involved in the regulation of redox balance, and they use Sec as the active site of its catalytic function [29,30]. Several decades ago, it was reported that selenium deficiency significantly caused the reduction of TrxR and GPx activities in experimental animals [31,32]. Based on the important role of TrxR and GPx in the regulation of redox balance, it is not difficult to find that selenium deficiency can increase the sensitivity of cells to oxidative stress [33,34].

The effects of selenium on cisplatin-induced nephrotoxicity have been widely investigated, but the results are still contradictory. Functionalized selenium nanoparticles (Se@Trolox) [35] and tea polyphenol-functionalized selenium nanoparticles (Se@TE NPs) [36] indicated to be promising Se species with potential application in the prevention of cisplatin-induced nephrotoxicity. More importantly, selenium exhibited the protective effects on cisplatin-induced nephrotoxicity in cancer patients [37,38]. On the contrary, however, some researchers showed that selenium did not have the protective effects in adult Wistar rats [39]. Thus, it is not clear whether selenium supplementation definitely alleviates cisplatin-induced nephrotoxicity.

Our and other previous studies have demonstrated that cisplatin is a very effective and specific inhibitor of TrxR1 and the inhibition of TrxR in the kidney is correlated with its nephrotoxicity. The change of the cellular thiol-dependent redox environment regulated by TrxR1 and GSH is a major event in the adverse effects of cisplatin in the kidney [40]. Activities of TrxRs and GPxs are controlled by the selenium level intake, and thus contribute to regulating redox signals and maintaining normal cellular redox homeostasis [26]. However, whether the elevation of the activities of the two selenoenzyme-containing systems can protect the kidney is not known. In this study, we fed the mice with diets containing different levels of selenium to investigate whether changes in the activities of TrxRs and GPxs can affect cisplatin-induced nephrotoxicity.

## 2. Materials and Methods

### 2.1. Materials

Cisplatin (DDP) was purchased from Aladdin (Shanghai, China). NADPH and 5,5′-dithiobis-(2-nitrobenzoic acid) (DTNB) were obtained from Biosharp (Guangzhou, China). Aurothioglucose (ATG) was purchased from WAKO (Tokyo, Japan). Glutathione reductase (GR) and iodoacetamide (IAM) were purchased from Sigma-Aldrich (St. Louis, MO, USA). RIPA lysis buffer was obtained from Beyotime Biotechnology (Shanghai, China). Selenium-deficient (SD, 0.01 mg/kg), selenium-normal (SN, 0.21 mg/kg), and selenium-enriched (SE, 1.01 mg/kg) diets were manufactured by Medicience (Nanning, China). All other reagents were obtained from the Sigma-Aldrich Chemical Co. (St. Louis, MO, USA) or Fisher Scientific Ltd. (Waltham, MA, USA). The pure recombinant wild-type rat TrxR1 protein, and anti-Trx1 and anti-TrxR1 antibodies were obtained from IMCO Corporation, Stockholm, Sweden (www.imcocorp.se accessed on 1 January 2020). Other antibodies were obtained from Proteintech (Wuhan, China). C57BL/6j mice were provided by Ensiweier (Chongqing, China).

### 2.2. Animals and Drug Administration

The mice experiments in this study were approved by the local Animal Ethics Committee with permission number IACUC-20201007-02. To investigate the effects of dietary selenium status on DDP nephrotoxicity, 3-week-old weanling male C57BL/6j mice were randomly divided into three groups, including the SD, SN, and SE group (12 mice per group), and fed with enough water and diets containing different levels of selenium (SD, SN, and SE), with light for 12 h and dark for 12 h. After the treatment for 4 months, 12 mice in each group fed with diets containing different selenium were divided into 2 subgroups (6 mice per subgroup). The mice in each subgroup were then treated with 9 mg/kg DDP or saline via intraperitoneal injection, respectively. In the first experiment, the number of mice deaths after the administration of DDP was counted each day and used to calculate the survival rate of the mice in 8 days. In the second experiment, the mice were sacrificed 72 h after cisplatin administration. Then the blood samples were collected for the detection of aspartate aminotransferase (AST), alanine aminotransferase (ALT), creatinine (CREA), and blood urea nitrogen (UREA) levels, and kidney and liver tissues were collected for histological and immunofluorescence analysis, enzyme activity measurements, qPCR, and Western blotting analysis.

### 2.3. Serum AST, ALT, CREA, and UREA Detection

Mice serum was obtained through centrifugation at 2500× *g*, 4 °C for 20 min after the blood clotted. The AST, ALT, CREA, and UREA levels in serum were detected by an automatic biochemistry analyzer (Biobase, Wolfenbüttel, Germany, BK400). Before detection, the 50 µL serum was diluted to 200 µL with saline.

### 2.4. Histological Analyses

Kidney tissues were fixed in 4% (*w*/*v*) paraformaldehyde and subjected to Servicebio (Wuhan, China) for hematoxylin-eosin and periodic acid–Schiff staining to evaluate kidney damage.

### 2.5. Western Blotting

Fresh kidney tissues (50 mg) were washed with cold 0.9% physiological saline and homogenized with 500 μL lysis buffer containing RIPA and 1 mM phenylmethanesulfonyl fluoride (PMSF). After centrifugation at 13,000× *g*, 4 °C, 10 min, the supernatants were collected and stored at −80 °C. Protein concentration was detected by a BCA kit (Sangon, Shanghai, China) according to the manufacturer’s instructions. After that, the kidney extracts were separated by 15% SDS-PAGE with a 25 μg protein sample loaded into each well. After electrophoresis, the proteins were transferred to the PVDF membrane. Subsequently, the membranes were blocked with 10% skim milk for 2 h at room temperature and incubated with anti-Trx1 (1:1000 dilution); anti-Trx2 (1:10,000 dilution); anti-TrxR1 (1:5000 dilution); anti-TrxR2 (1:5000 dilution); anti-Prx1 (1:2000 dilution); anti-Prx2 (1:2000 dilution); anti-Prx3 (1:2000 dilution); anti-GAPDH (1:5000 dilution); and anti-Hmox1 (1:2000 dilution) at room temperature for 1 h, or 4 °C overnight. Then, the membranes were incubated with corresponding goat anti-rabbit (1:5000 dilution) and goat anti-mouse (1:5000 dilution) secondary antibodies. Finally, the membranes were incubated with ECL reagents and the protein bands were detected using a chemiluminescence imaging system (6000, Clinx, Shanghai, China).

### 2.6. Measurement of TrxR Activity and GSH Level

TrxR activity was detected by a modified DTNB method in 96-well plates [40,41]. Briefly, 50 mg fresh kidney tissue was added to 500 µL PBS (PH 7.4), containing 1 mM PMSF. A total of 25 μg of protein was incubated with 100 μM NADPH for 5 min, with reference wells in which 300 nM ATG was added to inhibit TrxR activity specifically. Then, a reaction solution containing 100 μM NADPH and 2 mM DTNB was added. The increase in absorbance at 412 nm was recorded for 5 min with 1 min intervals. All experiments were performed at room temperature. TrxR activity was represented by the slope of the increase in absorbance over a minute and the certain activity value was calculated via the subtraction of the data from the values of reference wells.

The method to detect the GSH level also followed the modified DTNB method [40,41]. In brief, 50 mg fresh kidney extract was added to 500 µL 5% 5-sulfosalicylic acid and homogenized, followed by a centrifugation at 13,000× *g* for 10 min. The collected supernatants were diluted 10 times with TE buffer for further analysis. Subsequently, 5 μL of kidney extracts were incubated with 200 μM NADPH and 50 nM GR for 5 min. Then, a reaction solution containing 2 mM DTNB was added to measure the GSH level in the extracts. The other steps were performed in the same way as the detection of TrxR activity. In reference wells, additional GR was not added. All samples were measured at least three times.

### 2.7. Detection of GPx Activity

GPx activity was detected using H_2_O_2_ as substrate, according to the previous report [42]. In brief, 5 µL of 1 mg/mL protein sample or 5 µL serum samples were placed in the 96-well plate, and PBS was set as the reference. Then, a 100 µL GPx working solution containing 1 U/mL GR, 200 µM NADPH, 2 mM GSH, and 11.1 mM NaN_3_ was added. After the incubation at room temperature for 5 min, 100 µL of 2 mM H_2_O_2_ was added to start the reaction, whereas the absorbance at 340 nm was followed for 5 min. The activity of GPx was represented by the slope of absorbance versus time. All samples were measured at least three times.

### 2.8. Detection of Elements Content

Kidney or liver tissues (100 mg) were digested with 5 mL nitric acid and perchloric acid mixture (V nitric acid: V perchloric acid = 4:1) at room temperature overnight. Then, the acids were removed by heating. After cooling, 1 mL deionized water and 1 mL concentrated hydrochloric acid were added and the samples were heated and digested again. After the digestion, the acid was removed until the white smoke was completely drained. Finally, the samples were transferred to a volumetric bottle and distilled water was added, up to 5 mL. To measure the metal ion content in the serum, 0.05 mL of serum was picked up and the digestion steps were as described above. The selenium amount was determined by ICP-MS (NexION 10000G PE, Sykesville, MD, USA) [43].

Determination of calcium, magnesium, and iron content in kidney extracts was performed as follows: 0.5 mg kidney-extracted proteins were digested, and then the content of the element in the samples was measured according to the above-described procedures.

### 2.9. Real-Time Quantitative PCR

Total RNA of a kidney was extracted by the FastPure Cell/Tissue Total RNA Isolation Kit (Vazyme, Nanjing, China). Reverse transcription was performed for obtaining cDNA, and the mRNA level was detected by corresponding primers (Table 1). The gene ID numbers of targeted genes were obtained from the NCBI database and the exon spanning primers were designed by Primer Bank.

### 2.10. Statistical Analysis

Data were analyzed using an SPSS statistical package and performed by GraphPad Prism 8.0, shown as mean ± SEM. The difference among groups was analyzed by a one-way analysis of variance (ANOVA) or two-way ANOVA. A value of *p* < 0.05 was considered to be statistically significant.

## 3. Results

### 3.1. Dietary Selenium Altered Serum and Kidney Selenium Levels

**Figure 1:**
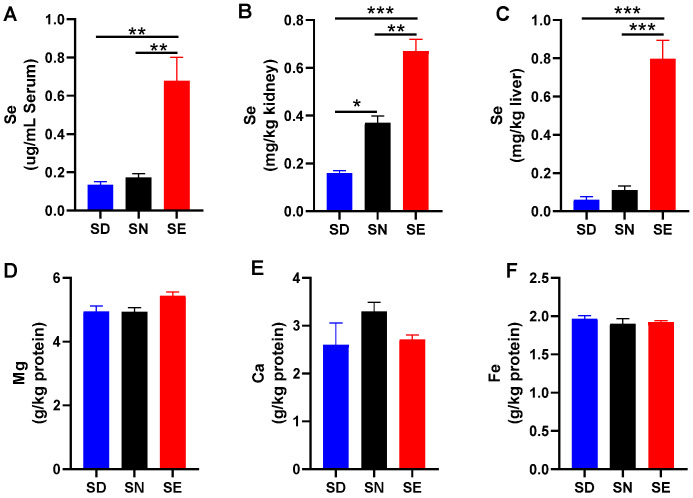
Selenium levels in serum (**A**), kidney (**B**), and liver (**C**), and magnesium (**D**), calcium (**E**), and iron levels (**F**) in kidney from the mice fed with diet containing various amounts of selenium. Values were presented as mean ± SEM; *, *p* < 0.05; **, *p* < 0.01; ***, *p* < 0.001; one-way ANOVA, *n* = 3. SD, selenium-deficient; SN, selenium-normal; SE, selenium-enriched.

To establish mice models that were selenium-deficient (SD), selenium-normal (SN) and selenium-enriched (SE), the weanling mice were fed with SD, SN, and SE diets for 4 months. Subsequently, we measured selenium levels in serum, kidney, and liver tissues using ICP-MS. The serum selenium level in the SE group was higher than that in SD and SN groups, whereas there was no significant difference in the selenium levels in the serum in SD and SN groups (Figure 1A). The selenium level in kidney and liver from the mice of the SE group also had a significant (*p* < 0.05) increase compared to those of the other two groups (Figure 1B,C). It indicated that feeding with an SE diet caused selenium levels to increase in the serum, kidney, and liver, and selenium levels in the kidney decreased after feeding mice with an SD diet. We also detected calcium, magnesium, and iron levels in kidney extracts, and all the elements’ levels did not have significant changes (Figure 1D–F), which implied that different levels of a selenium diet did not affect the contents of other metal ions in the kidney.

### 3.2. Selenium Status Affected Cisplatin-Induced Nephrotoxicity

**Figure 2:**
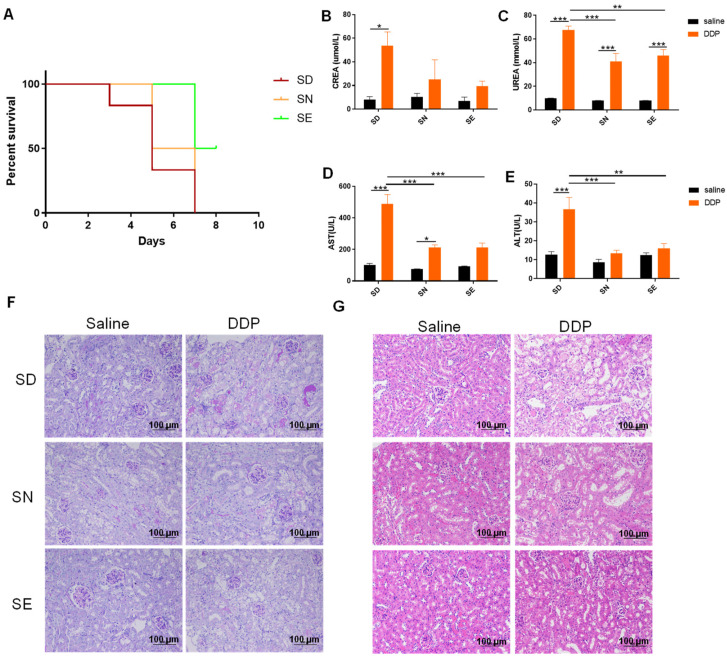
Selenium status in diet affects mice nephrotoxicity caused by DDP. (**A**) Survival curve of mice treated with 9 mg/kg DDP (*n* = 6); (**B**,**C**) serum CREA, UREA levels 72 h after DDP administration (*n* = 4–6); (**D**,**E**) serum AST, ALT levels 72 h after DDP administration (*n* = 5–6); (**F**,**G**) kidney tissues were stained and observed via periodic acid–Schiff staining and hematoxylin-eosin staining 72 h after DDP administration, magnification 200×, scale bar 100 µm, *n* = 3. A representative example was shown. Data were presented as mean ± SEM; * *p* < 0.05; ** *p* < 0.01; *** *p* < 0.001; two-way ANOVA. SD, selenium-deficient; SN, selenium-normal; SE, selenium-enriched; DDP, cisplatin.

To detect the effect of selenium in diet on the cisplatin-induced nephrotoxicity, mice fed with diets containing different levels of selenium were treated with 9 mg/kg DDP. The mice survival curves are shown in Figure 2A. The number of mice that died within 8 days was recorded. Mice in the SD group started to die 3 days after DDP treatment. All mice in SD and SN groups were dead on the eighth day, whereas half of mice in the SE group were still alive. This indicated that the supplementation of selenium in diet played a protective role against cisplatin-induced damage.

After four months of feeding, the body weight of mice in each group increased to about 35 g. Subsequently, three days after DDP treatment the body weight of mice in all SD, SN, and SE groups was significantly reduced to about 30 g. In contrast, the body weight of the mice without cisplatin treatment did not change, and there was no significant difference in body weight in the groups of different diets (data not shown). To evaluate the tissue damage caused by DDP, we measured the markers of mice kidney and liver damage. There was a significant increase in CREA, UREA, AST, and ALT levels in the SD group 72 h after DDP administration (Figure 2B–E). These results suggested that treatment with DDP induced more severe nephrotoxicity and hepatoxicity in the selenium-deficient group. PAS staining (Figure 2F) was used to detect the formation of purplish-red glycogen-related complexes to indicate the injury of the kidney. More severe damage occurred in the kidney of mice fed with a selenium-deficient diet after DDP exposure, and selenium supplementation could significantly reduce the injury. In addition, HE staining (Figure 2G) showed that the kidney from the mice in the SD group had obvious damage, presented as a large number of cortical renal tubules dilated with irregular shape, and epithelial degeneration. Taken together, these results demonstrated that kidney injury induced by DDP in mice was affected by the status of selenium in diet.

### 3.3. Increased Selenoenzyme Activity Mediated Oxidative Stress

TrxRs and GPxs are the selenoenzymes in the Trx and GSH antioxidant systems. To explore whether selenium in diet changed TrxR and GPx activities, we used a modified DTNB method to detect the TrxR activity and GSH level, and also used H_2_O_2_ as a substrate to detect the GPx activity in the kidney and serum.

**Figure 3:**
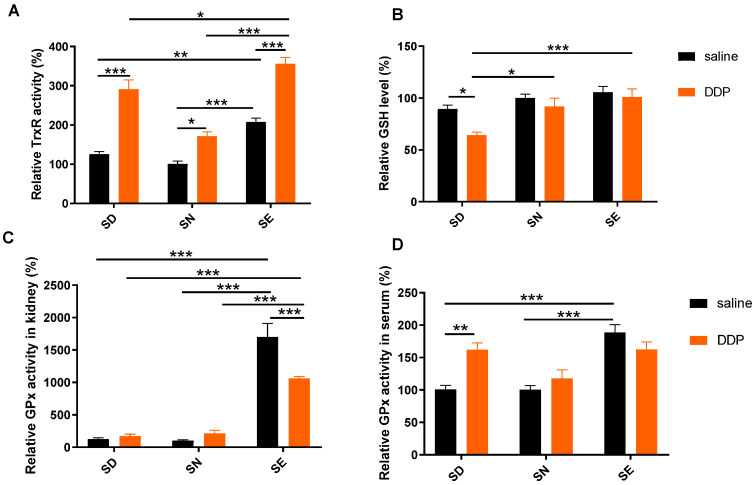
Selenium status affects TrxR and GPx activity and GSH levels in kidney after DDP treatment. (**A**) Total TrxR activity and (**B**) GSH levels in kidney were measured using the DTNB assay. Total GPx activity in kidney (**C**) and serum (**D**) were measured using H_2_O_2_ as a substrate to detect. Each sample was analyzed in triplicate. Data are normalized using the value from samples of SN saline group as control (100%) and presented as mean ± SEM; * *p* < 0.05; ** *p* < 0.01; *** *p* < 0.001; two-way ANOVA, *n* = 5–6. SD, selenium-deficient; SN, selenium-normal; SE, selenium-enriched; DDP, cisplatin.

**Figure 4:**
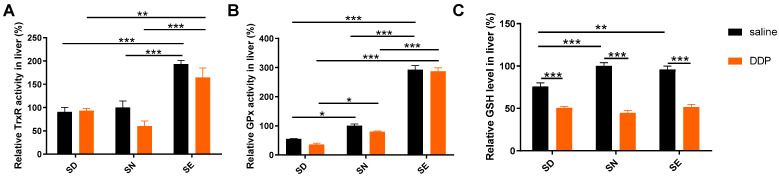
Selenium status affects liver TrxR and GPx activity and GSH levels after DDP treatment. (**A**) Liver TrxR activity, (**B**) GPx activity, and (**C**) GSH levels. Each sample was analyzed in triplicate. Data are normalized using the value from samples of SN saline group as control (100%) and presented as mean ± SEM; * *p* < 0.05; ** *p* < 0.01; *** *p* < 0.001; two-way ANOVA, *n* = 5–6. SD, selenium-deficient; SN, selenium-normal; SE, selenium-enriched; DDP, cisplatin.

Feeding with a selenium-enriched diet enhanced TrxR and GPx activities in the kidney (Figure 3A,C) and GPx activity in the serum (Figure 3D), although the activity of TrxR increased in all groups 72 h after DDP exposure (Figure 3A). It indicated that selenoenzyme activities in animals increased with an SE diet, which may be beneficial for the regulation of oxidative stress in mice, thus alleviating cisplatin-induced nephrotoxicity. The level of kidney GSH decreased in the SD group after the cisplatin exposure, whereas the GSH level of the kidney in SN and SE groups did not have obvious changes (Figure 3B). These results suggested that the increase in selenoenzyme activity may be an important event to fight against oxidative stress induced by cisplatin in mice in the SE group. The decrease in the GSH level of mice in the SD group after cisplatin administration indicated that the oxidative stress is more intense, resulting in more serious kidney damage. In addition, a selenium-enriched diet could increase selenoenzyme activities and the GSH level in the liver, thus reducing cisplatin-induced hepatotoxicity (Figure 4).

### 3.4. Effect of Selenium Level on Redox Regulation

**Figure 5:**
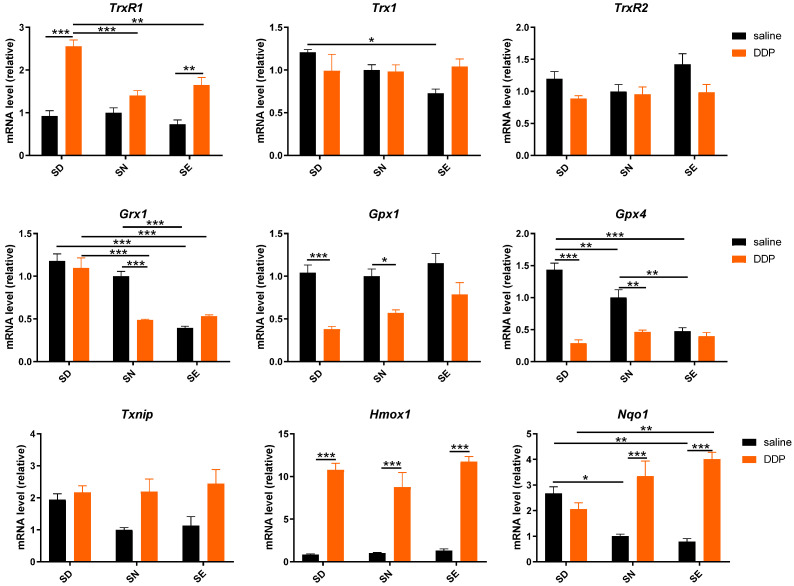
mRNA level of antioxidant enzymes in mouse kidney following the treatment with DDP. mRNA level of GAPDH was used as the control, related mRNA levels were shown as 2^−(ΔΔCt)^. Data are normalized using the value from samples of SN saline group as control (=1) and presented as mean ± SEM; * *p* < 0.05; ** *p* < 0.01; *** *p* < 0.001; two-way ANOVA, *n* = 4. SD, selenium-deficient; SN, selenium-normal; SE, selenium-enriched; DDP, cisplatin.

To have a further understanding of the change in the redox environment mediated by TrxR and GPx, we used RT-qPCR and Western blotting to measure and compare the mRNA and protein levels of Trx and GSH systems and some Nrf2-mediated genes in the kidney. Notably, the mRNA level of TrxR1 in the SD group was dramatically elevated and the mRNA level of GPx1 and GPx4 in the SD and SN groups decreased significantly after cisplatin exposure. In contrast, the mRNA level of TrxR1 was increased and GPx1 and 4 did not have significant changes (Figure 5). This result indicated that more extensive changes for the selenoprotein genes involved in the defense against oxidative stress occurred in SD and SN groups.

**Figure 6:**
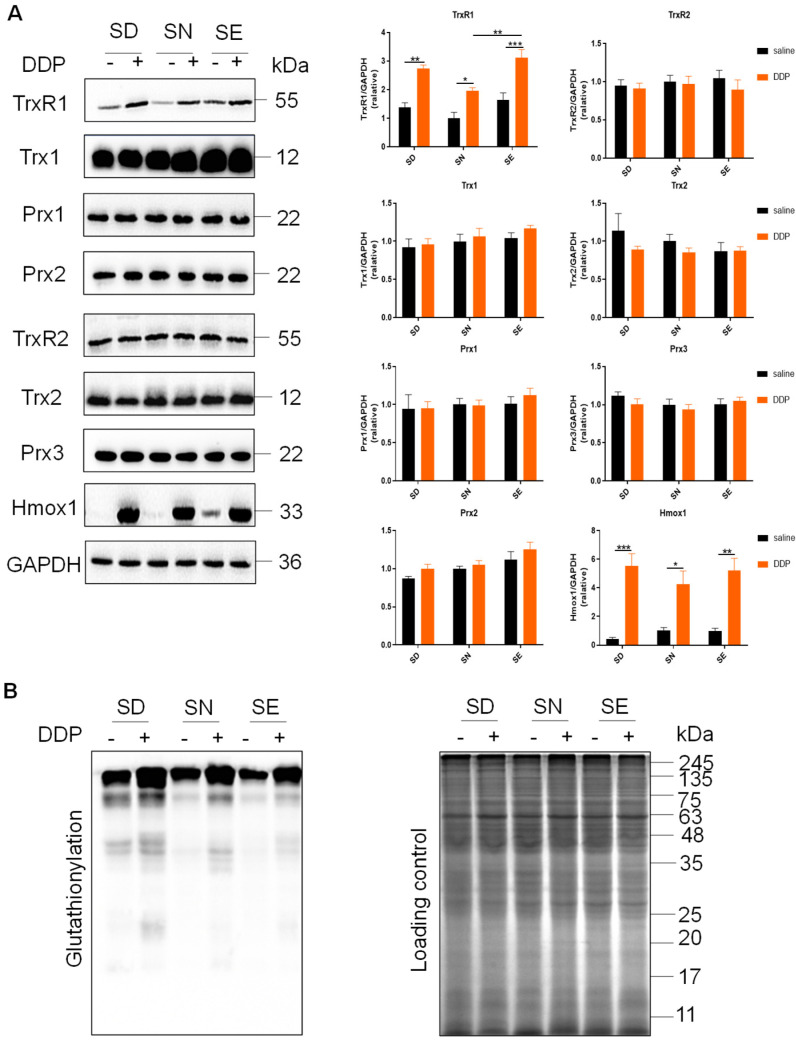
Effects of selenium status on redox-related protein expression and glutathionylation level upon DDP treatment. (**A**) Cytosolic and mitochondrial protein expression upon DDP treatment. Data were normalized by comparing the protein levels of targeted group with those in selenium-normal mice treated with saline (=1). (**B**) Protein glutathionylation within mice kidney after DDP treatment. The CBB-stained gel was used as a loading control, n = 4. CBB, Coomassie brilliant blue. Data are shown as mean ± SEM; * *p* < 0.05; ** *p* < 0.01; *** *p* < 0.001; two-way ANOVA, n = 4. SD, selenium-deficient; SN, selenium-normal; SE, selenium-enriched; DDP, cisplatin.

Further analysis also showed the protein expression was consistent with the mRNA levels (Figure 6A). The protein levels of TrxR2, Trx2, and Prx3 and mRNA levels of TrxR2 did not have obvious changes (Figure 6A), indicating that the Trx system in the mitochondrial matrix may not be significantly affected by DDP treatment. Both mRNA and protein levels of TrxR1 and Hmox1 were elevated, which indicated that the activation of Nrf2 regulation occurred after DDP exposure. This result was consistent with our previous results that showed Nrf2 activation in kidney tissue [40]. Meanwhile, the elevation of Nqo1 was blocked in the SD group 72 h after DDP administration, indicating that the activation of the Nrf2 antioxidant regulation pathway might be affected. Furthermore, the levels of glutathionylation were significantly increased after DDP treatment, and selenium supplementation reduced the increase as compared to the SD group (Figure 6B), suggesting that the level of renal oxidative stress induced by cisplatin was correlated with the kidney selenium level.

## 4. Discussion

Nephrotoxicity is the most serious adverse effect of cisplatin [12,13,44,45,46], and currently, effective approaches to relieve it are limited. The supplementation with selenium is an interesting topic for alleviating DDP-induced toxicity, but thus far it is still inconclusive [37,38,39]. Notably, most of the clinical studies are positive, in particular in the cases of long-term usage of selenium. One example is a study that examined the effects of the selenium supplementation on the adverse effects in patients with ovarian cancer undergoing chemotherapy including cisplatin in combination with cyclophosphamide. After 2 and 3 months of supplementation with selenium, the adverse effects including nausea, vomiting, stomatitis, hair loss, flatulence, abdominal pain, weakness, and loss of appetite were all alleviated [47]. This result is in agreement with a recent investigation to determine the predictors in compliance with DDP treatment for locally advanced head and neck squamous-cell carcinoma. Selenium deficiency before treatment is an important negative factor for the usage of full-dose cisplatin [48]. All these are consistent with our results here that the selenium status in diet affected DDP-induced nephrotoxicity. However, to our knowledge there are no reports about the influence of selenium status in diet or serum on the general survival of cancer-bearing patients, which should be an interesting topic based on the above mentioned and our results here.

Oxidative stress is known to be a major mechanism in cisplatin-induced nephrotoxicity [19,49], but no useful drug target has been verified to alleviate the toxicity caused by the oxidative stress. Our previous study has demonstrated that TrxR activity in a mice kidney at the early stage (6 h after DDP exposure) was significantly inhibited by DDP. Subsequently, TrxR activity was recovered together with the increase in TrxR1 mRNA and protein levels 24 h after DDP exposure, which may be due to the activation of Nrf2. The elevation of Nrf2-mediated antioxidant genes increased the cellular thiol level to achieve redox balance, but the cellular thiol-dependent redox environment regulated by TrxR1/Trx1 and GSH/Grx continues to move towards an oxidizing state. Notably, 72 h after the DDP administration, the thiol level went back to the basic level, but the cellular redox state became oxidized and correlated with cisplatin-induced nephrotoxicity [40]. This raises the question of whether TrxR1 is a useful druggable target for alleviating the DDP-caused adverse effects. As TrxR1 is a selenoprotein, we thus explored feeding mice with different levels of selenium to change the activity of selenoproteins in renal cells in order to investigate whether changes in the redox microenvironment caused by selenoprotein activity can affect cisplatin-induced nephrotoxicity.

Selenium status in diet does not only affect the activities of TrxR, but also the other selenoproteins. Here we demonstrated clearly that feeding the mice with a diet containing various levels of selenium for four months resulted in different levels of selenium in the serum, kidney, and liver, and various levels of TrxR and GPx activities. This provided a useful model to investigate the relationship between selenium, selenoproteins, and DDP-induced toxicity. The mice survival curve and markers for the tissue damage confirmed that selenium supplementation was beneficial for the alleviation of DDP-induced nephrotoxicity. Moreover, an increase in TrxR and GPx activity and thiol redox environment regulation capacity had a close link with the reduction of DDP-induced toxicity. These results strongly suggested that TrxR1 is a potential drug target and selenium supplementation may be a viable method for the alleviation of DDP-induced toxicity.

In this study, 72 h after cisplatin administration was selected to be the suitable time point for analysis. The choice was made with reference to the selection that most researchers use to analyze the toxicity of cisplatin [50,51,52,53,54,55]. Moreover, our previous work analyzed the toxicity at different time points of cisplatin administration, and found that the toxicity became severe and the protein glutathionylation level had a significant increase in the kidney at 72 h [40]. Here we would like to evaluate the effects of selenium status in diet on the change of the selenoproteins, cellular redox environment, and the toxicity at the time point. Interestingly, there were some difference between the kidney and liver in the TrxR activity changing trend after the treatment of DDP. The TrxR activity increased in the kidney, whereas the TrxR activity in the liver had a slight decrease (Figure 3 and Figure 4), which is consistent with a previous observation [40]. The enzymatic method used here is well characterized using a 300 nM ATG as a specific inhibitor of TrxR. The difference in the TrxR activity changing trend in different tissues should not be due to the accuracy of the analytical method. However, it also does not mean that one compound had different initial effects on one enzyme in different tissues. One possible explanation is that the enzymatical analysis was performed for the samples obtained from the mice treated with DDP for 72 h and, at this time point, redox compensation regulation in the tissues already occurred as described in the reference [40]. Therefore, the difference in cellular compensation regulation may account for the resulting difference in the TrxR changing trend in the liver and kidney.

Besides that, the increased selenoenzymes activity is an important reason for the resistance of DDP-induced oxidative stress in SE group mice, as the level of GSH decreased in the SD group, suggesting that selenium deficiency may lead to more serious oxidative stress in the kidney. What is more, the levels of glutathionylation in the SD group were significantly increased after DDP treatment, whereas in the SE group there was a significant improvement, which also confirmed that the thiol redox environment is a key factor to determine the DDP-induced nephrotoxicity.

There are several limitations to this study. One limitation is that only the male mice were used for investigation. Previous studies have indicated that the gender difference may result in a difference in the cisplatin nephrotoxicity [56,57,58]. To avoid the effects of the gender factor on the result, we only selected the male mice in this study. Thus, we cannot confirm the role of gender difference in cisplatin nephrotoxicity nor clarify whether the gender-resulted difference is related to thiol redox regulation. Another limitation is that we have not studied whether the selenium status in diet affects the therapeutic efficiency of DDP against the cancer. If selenium supplementation decreases adverse effects and enhances the therapeutic activity at the same time, the cisplatin plus selenium will become a useful approach for cancer therapy.

## 5. Conclusions

Our results demonstrated that the activities of the selenoproteins TrxR and GPx played an important role in the nephrotoxicity of cisplatin. Selenium deficiency induced more intense oxidative stress, and selenium supplementation could alleviate cisplatin-induced nephrotoxicity. Selenoprotein TrxR is a key protein that has a selective interaction with cisplatin, which may be used as a drug target for alleviating cisplatin nephrotoxicity.

## Figures and Tables

**Table 1:** Primer sequences used in the study with real-time quantitative PCR.

No.	Gene	Sequence (F)	Sequence (R)
GI: 123701799	*Trx1*	CATGCCGACCTTCCAGTTTTA	TTTCCTTGTTAGCACCGGAGA
GI: 110224446	*TrxR1*	CCCACTTGCCCCAACTGTT	GGGAGTGTCTTGGAGGGAC
GI:1212477963	*TrxR2*	GATCCGGTGGCCTAGCTTG	TCGGGGAGAAGGTTCCACAT
GI:1335348593	*Grx1*	GCTCAGGAGTTTGTGAACTGC	AGAAGACCTTGTTTGAAAGGCA
GI:1043678440	*GPx1*	AGTCCACCGTGTATGCCTTCT	GAGACGCGACATTCTCAATGA
GI:1547242143	*GPx4*	GCCTGGATAAGTACAGGGGTT	CATGCAGATCGACTAGCTGAG
GI: 254553443	*Txnip*	TCTTTTGAGGTGGTCTTCAACG	GCTTTGACTCGGGTAACTTCACA
GI: 195947362	*Hmox-1*	AAGCCGAGAATGCTGAGTTCA	GCCGTGTAGATATGGTACAAGGA
GI: 161621259	*Nqo-1*	AGGATGGGAGGTACTCGAATC	AGGCGTCCTTCCTTATATGCTA
GI: 576080554	*GAPDH*	AGGTCGGTGTGAACGGATTTG	TGTAGACCATGTAGTTGAGGTCA

## Data Availability

The data presented in this study are available in the paper.
